# Neurasthenia and Nasal Disease1Read before the Buffalo Medical Club, October 15, 1890.

**Published:** 1891-01

**Authors:** Frank Hamilton Potter

**Affiliations:** Lecturer on Diseases of the Nose and Throat in the Medical Department of Niagara University; Surgeon to the Nose and Throat Department Erie County Eye, Ear, and Throat Infirmary; 273 Franklin Street


### NEURASTHENIA AND NASAL DISEASE.[1]

By FRANK HAMILTON POTTER, M. D.

Lecturer on Diseases of the Nose and Throat in the Medical Department of Niagara University; Surgeon to the Nose and Throat Department Erie County Eye, Ear, and Throat Infirmary.

THIS subject was selected in the first place because there has recently been some discussion upon it, in the course of which very divergent views were expressed; and in the second place, because it will serve to give an opportunity to state some of the writer's opinions concerning the relation between certain constitutional diseases and disease of the upper air tract. Let us first examine the evidence bearing on the dependence between Neurasthenia and Nasal Dis-

1. Read before the Buffalo Medical Club, October 15, 1890.

ease. In 1889, Dr. Wm. H. Daly, of Pittsburg, read a paper before the American Laryngological Association, in which he held that many times chronic disease of the nasal passages preceded or was at least concomitant to the condition expressed by the term Neurasthenia. He presented two cases in detail, illustrating this view, but referred in all to ten cases occurring in his practice. His summary of this evidence was to the effect that in all of these cases there was obstinate inflammatory aphonia, attended with utter prostration, nervousness, and insomnia; seven of these patients had nasal disorder of a chronic character; the remaining cases all had as leading factors naso-pharyngo-laryngeal disease in some form, with neurasthenia in some form also. He then adds, that while one swallow does not make a summer, a flock of them will cause us to look for a change of weather: and will not this little flock of cases cause us to look out for this too widely prevailing constitutional condition, as one of the unfortunate possibilities or concomitants of disease of the upper air tract? That they coëxist, there can be no question.

In the discussion on this paper two opinions were expressed. Dr. Roe and Dr. Sajous supported Dr. Daly, maintaining that the constant nagging of a local irritation would produce, sooner or later, a depressed condition of the nervous system; Dr. Hinkel and Dr. Langmaid believed that while the local disease could be a factor, it was only one of many — not the main—causes, and that the removal of the local disease would frequently fail to relieve the neurasthenic conditions.

Here, then, we have two divergent views upon this question. The issue is joined; which is correct? But this is not all, — the question is not yet fully stated. The year after Dr. Daly read his paper, Dr. W. F. Chappel, of New York, published a paper entitled Neurasthenia and Neuralgia from Traumatism of the Nasal Passages.[1] This paper was enough to startle the most enthusiastic rhinologist. Dr. Chappel related cases showing that neurasthenia, neuralgia, and peripheral neuritis sometimes resulted or followed upon operations upon the nasal passages. He admitted that these cases might have been neurotic, but they exhibited none of the symptoms of the above-named diseases until after the intranasal operations. He, therefore, concludes that "operations in the nasal passages should not be undertaken lightly, and with the impression that if they don't do any good, they will not be baneful; also

1. New York Medical Record, May 10, 1890.

that great experience and judgment may be required to determine the cases suitable for operation, and likely to be benefited thereby. . . . . . . If the patient is very neurotic, or gives the history of having suffered from what is called nervous prostration, although the physical conditions might be excellent, I would discourage operative measures, unless imperatively demanded."

This view of the subject is, in our opinion, often lost sight of altogether. It should make us pause and reconsider the reasons for, and perhaps the methods of, performing intra-nasal operations. Dr. Chappel has been supported in rather vigorous language in a recent editorial in *The Journal of Laryngology and Rhinology* for June, 1890. One or two remarks from that article will indicate its position. "The causes of neurasthenia, obscure as they are, are not to be sought in some slightly abnormal condition of an organ such as the nose, and to imagine that local treatment of this, or indeed of any individual organ, is going to reward us with successful therapeutic results, is to subject ourselves to such failures as Dr. More Madden very honestly has recorded. . . . . . It seems to us that to draw the attention of such a patient off general treatment, and to concentrate it upon one organ, even if that be only the nose, is to put him in worse condition than when we started. . . . . . It would be a pity if rhinology were to incur the reproach of a 'narrow specialism,' and to fall under a ban similar in this respect to a narrow-minded gynecology. . . . To remove a vital cause of irritation is right and proper, but to assert that the slight pathological abnormalities met with in many nasal organs, even where they are accompanied with nasal catarrhs, is in real proportion of neurasthenic individuals a potent cause of their troubles, is, we think, to take up an untenable position."

This article overdoes the matter, and swings too far in the opposite direction. It is not necessary to support the position maintained by the author to under-estimate the value of any factor that may enter into the production of the disease under discussion. The expressions, "only the nose," and "slight pathological abnormalities," are out of place, and lessen the force of the argument. It, however, serves to show how radically different views can be held upon this question.

While this is not, by any means, all of the literature on the subject, it is not necessary to quote further,— it is more important and bears directly upon the management of these cases, to ascertain, if possible, the truth concealed in the opposing views.

In the discussion of diseases of nervous origin, or, at least, exhibiting for the most part nervous phenomena, it is very difficult for some writers to avoid extremes of statement. With them one swallow does indeed seem to make a summer. Specialists have incurred criticism by the narrowness exhibited in an argument to maintain a very sound proposition. It is true, however, that their vehemence has been aggravated by the reluctance of the general profession to accept their views, even when demonstrated to be correct. It is difficult to tell which is the safer leader, a specialist or a generalist, when both refuse to see the light.

It has been shown beyond a reasonable doubt that such diseases as chorea, epilepsy, asthma, hay-fever, neurasthenia, the various forms of neuralgia, etc., are sometimes due to disease of the nasal passages. To claim that they are always or even generally due to pathological changes within the nose, is fanciful and extreme. The percentage, however, is sufficiently marked to warrant the statement that in every case of the diseases referred to, the upper air passages should be thoroughly examined in order not to overlook the possibility of a nasal element as one of the causative factors.

As far as neurasthenia is concerned, I believe I have seen cases illustrating both extremes, as detailed above. In the first place, two instances occurring in my own practice, and three or four cases pointed out to me by others as occurring in their practice, is convincing as to the statement that neurasthenia may follow upon surgical interference with the nasal passages. The case which first called my attention to this possibility is worth mentioning. A woman, about thirty-five years of age, presented herself with marked nasal obstruction. This was due to an irregular septum from which into each nostril a spur projected. It was also complicated by marked hypertrophy of the soft parts. The secondary symptoms, due to pressure between the walls of the nasal passage, the blocking up of the secretions, and the continual mouth-breathing, were very marked. The removal of the obstructive lesions by surgical means was advised and consented to. The septal spurs were first removed, and at that time it was the intention to remove the hypertrophies also. This was abandoned afterwards for sufficient reasons. Following close upon the removal of the second spur, the patient began to complain of pain and constriction about the head; she had great depression of spirits and hopelessness about her case and about herself generally. She was unable to perform her duties; was constantly dreading something new, and was indeed in a most unfortunate condition. Local treatment, excepting of the mildest kind, was abandoned, and attention was directed to restoring her general health. She was under observation about a year. Her health finally returned, though it was a hard fight and an experience I do not wish to repeat. Another year has elapsed since treatment was discontinued, and she still remains well. The local condition is fairly good, nasal respiration having been partially reëstablished.

I still think the nasal treatment was necessary, though it might have proceeded with less energy. One observation occurs to me, bearing not only upon this case but upon others of a similar nature. May not the constant irritation of an obstructive nostril with the associated symptoms, direct and indirect, so influence the nervous system as to cause an explosion, so to speak, on the additional disturbance of an operation ? Is it fair to condemn nasal surgery when this result occurs, as these cases are generally of long standing with a nervous system abnormally sensitive ?

The second case need not be detailed, as it was not so well marked and was more easily controlled. I think the experience of the first case was of advantage as to this one.

The other class of cases, namely, those in which the neurasthenic symptoms disappear after nasal respiration has been restored, are more pleasant to contemplate and fortunately much more numerous. It is of this class of cases that authors speak with such positiveness ; and, indeed, we cannot blame them, as the results are brilliant ; only the reverse side of the picture should be always in mind. Now that John N. Mackenzie has reminded us that we are told in the Book of Genesis that when God made man it was not into his mouth, but into his *nostrils*, that he breathed the breath of life, we will perhaps pay more deference to this truth of Scriptural physiology. It is not remarkable that when this "breath of life" fails to obtain entrance by its natural channel, that disastrous consequences result; or that, in a certain proportion of these cases, the symptoms of such failure are expressed by disturbances of the nervous system.

The nose is concerned in olfaction, respiration, audition, and voice-production, and when obstructed these functions are imperfectly performed. Add to this the contact that usually exists between the walls of the nostrils in badly obstructed cases, thus bringing surfaces together that were meant to be separated, and we have all the conditions present sufficient to account for the nervous phenomena sometimes seen. Whatever the explanation, the clinical observations remain, and justify the opinion that in a given proportion of cases the chief factor in the development of neurasthenia is obstruction to the upper air tract, and that the removal of this obstruction is the first step in the relief of the disease.

A few cases will illustrate this class of patients :

Case I. Dressmaker, aged 27, of nervous temperament, appetite and digestion fair, complains of pain about head and face, restless, suffers much from insomnia, is fearful that she must abandon her work, etc., hypertrophic rhinitis affecting both nostrils ; mild treatment at first combined with strychnia and iron internally ; no result ; then hypertrophies removed, from which time improvement is dated. The patient was seen a short time since after an interval of six months, and reported herself as well.

Case II. Mrs. ———, aged 43, markedly neurasthenic, gave the history of a long period of illness and treatment by many physicians. Had been a mouth-breather for a long time. Complained of fullness about the head, neuralgia of head and face, ringing in the ears. Had severe paroxysms of coughing ; was pale, weak, and despondent. She was sent to me with special reference to the stoppage of the nasal passages. They were found entirely filled with mucous polyps. These were removed slowly,— two or three at a time, every fourth day. Fifteen were taken out in all, and the appropriate after-treatment was continued for some time afterwards. The change was a very rapid one. She began to improve in weight, in color, and in strength. The neuralgia and ringing in the ears disappeared. The cough was more obstinate, but that also was finally controlled. Of course, the general supporting treatment was continued all this time, but it is to be noted that she had been receiving the latter for many months, and that real improvement began only after nasal respiration was reëstablished.

Case III. Railroad engineer, aged 35 years ; naturally of a strong constitution ; had suffered for the last two years from facial neuralgia and severe headache. Had lost so much in strength, weight, etc., that he was fearful that he must give up his situation. Is nervous, irritable, and despondent. The nose was almost occluded from deformity of the septum and hypertrophy of the soft parts. The pharynx and naso-pharynx were highly congested and sensitive, and the latter was covered with tenacious mucus. The hypertrophies were removed, the septal deformity corrected, and the patient made a good recovery.

Case IV. Clerk, aged 30 ; tall and pale, appetite poor digestion easily disturbed, nervous, and suffers from insomnia. Is confined at desk-work, and has very little exercise. Takes cold easily. Has pain and sense of fullness between and above the eyes. Complained of nasal obstruction, and said that he felt sure that he would feel better if he could breathe naturally. Was advised that the nasal condition, in all probability, was but one of a number of factors entering into his case, and on that understanding I consented to treat him. The right nostril was found filled with a large bony ridge running along the sutural line between the vorner below and the ethenoidal plate above and the triangular cartilage in frout. In the left nostril the middle turbinated bone was greatly enlarged and pressed firmly against the septum. There was, besides, as was to be expected, a chronic inflammatory state of the entire nasal mucous membrane. These obstructions were removed, and the parts treated in the usual manner. The relief was as great as in any case coming under my observation. General treatment was, of course, employed, the patient's diet, exercise, hours of rest, etc., carefully considered, and at the end of a few months he was discharged well. He always maintained, and in this confirmed my own judgment, that the starting point in his recovery was the restoration of nasal breathing.

Case V. Miss ———, aged 25, servant; family history excellent; personal history, with the detail of symptoms, would fill a volume; fairly well nourished; complexion sallow; says is always tired and does her work with difficulty; severe attacks of frontal headache; has recently lost considerable in weight, and has become very despondent; voice muffled; mouth breather; throat constantly filled with unpleasant discharge; pain in the ears, etc., etc. Examination showed the naso-pharynx filled with the so-called adenoid tissue. The nose seemed merely in a state of passive congestion as it became freer under the use of cocaine, and exhibited no deformity. The adenoid tissue was removed, the nose and throat treated for some time afterwards with cleansing washes and mild astringent solutions. General supporting treatment was also given. The patient is now well, the nervous symptoms have disappeared, and she is able to earn her living, which before treatment she was hardly able to do.

These, of course, are selected cases — the symptoms in them were marked, and the relief following treatment also marked. They have been selected with a view of showing some of the pathological conditions of the nose and throat that may cause or be associated with neurasthenia.

In view of the fashion in some quarters to speak of the nasal lesions seen in the class of cases just considered as "slight pathological abnormalities," it may be pertinent to inquire whether any lesion that interferes with the physiology of an organ should be characterized as "slight." A stricture of the urethra, for instance, may not be as *great* as an ovarian tumor, but it may, nevertheless, produce such disastrous consequences as to warrant operative interference. The same thing can be said of many diseases of the eye, and of the ear, and so on. I would not magnify these things unduly, nor would I be blind to their true significance. As far as nasal surgery is concerned, it seems to me we should be guided by two propositions: First, the restoration of nasal respiration; and second, the removal of any conditions causing contact or pressure between the walls of the nasal passages. In the application of these principles the bias of the individual must be taken into account. We have already learned that in the neurotic subject we must proceed with caution, and there may be many other lessons in store for us which we will have to learn through a more extensive experience. However, between those who would do nothing and those who would practise extravagant surgery, there must be a rational middle course which we ought to follow, especially when we are considering the relation of any one organ to any particular disease.

In conclusion, it will be observed that an attempt has been made to present the subject of the relation of nasal disease to neurasthenia from both sides, and to contend that all the disease seem sometimes to depend upon pathological changes in the nasal passages should be so studied. It would be unfortunate if, after all, the writer were to be placed among that class of physicians well represented by a gentleman in the West, who, I am informed, announces that he cures asthma by a simple process of "stretching the rectum." Such a statement betrays faulty methods, and deserves notice chiefly for the warning it conveys. We trust that in this instance the warning has been heeded, and that the results of our experience, as presented in this paper, will be confirmed by the observations of others.

273 Franklin Street.

---

Infants' Food. — Dr. Geo. B. Fowler, in an article recently published in the *Medical Record*, gives the preference in most cases to the following easily prepared food: "Put four tablespoonfuls of rice into three pints of water and boil half an hour; then set aside on back of range to simmer during the day, water being occasionally added by the cook to maintain the original three pints. At night strain through a colander and place on ice. When cold a paste is formed. Three tablespoonfuls of this paste are added to each nursing bottle (half pint) of milk, and fed during the next day, a fresh supply of rice paste being under way in the meantime. Should there be constipation, I use farina, prepared in the same way, and used in the same proportion. Rice is astringent, farina laxative."— *Medical Age.*

